# A Vessel Active Contour Model for Vascular Segmentation

**DOI:** 10.1155/2014/106490

**Published:** 2014-07-01

**Authors:** Yun Tian, Qingli Chen, Wei Wang, Yu Peng, Qingjun Wang, Fuqing Duan, Zhongke Wu, Mingquan Zhou

**Affiliations:** ^1^College of Information Science & Technology, Beijing Normal University, Beijing 100875, China; ^2^Business School, Henan Normal University, Xinxiang 453007, China; ^3^Department of Obstetrics and Gynecology, Navy General Hospital, Beijing 100048, China; ^4^School of Design, Communication & Information Technology, University of Newcastle, Callaghan, NSW 2308, Australia; ^5^Department of Radiology, Navy General Hospital, Beijing 100048, China

## Abstract

This paper proposes a vessel active contour model based on local intensity weighting and a vessel vector field. Firstly, the energy function we define is evaluated along the evolving curve instead of all image points, and the function value at each point on the curve is based on the interior and exterior weighted means in a local neighborhood of the point, which is good for dealing with the intensity inhomogeneity. Secondly, a vascular vector field derived from a vesselness measure is employed to guide the contour to evolve along the vessel central skeleton into thin and weak vessels. Thirdly, an automatic initialization method that makes the model converge rapidly is developed, and it avoids repeated trails in conventional local region active contour models. Finally, a speed-up strategy is implemented by labeling the steadily evolved points, and it avoids the repeated computation of these points in the subsequent iterations. Experiments using synthetic and real vessel images validate the proposed model. Comparisons with the localized active contour model, local binary fitting model, and vascular active contour model show that the proposed model is more accurate, efficient, and suitable for extraction of the vessel tree from different medical images.

## 1. Introduction 

Vessel segmentation in images from different modalities is critical for medical diagnosis assistance as well as treatment and surgery planning. It is a key step for quantification of pathology. Although a large quantity of past and ongoing dedicated research on this topic exists, vessel segmentation remains a challenging task because of intensity inhomogeneity and weak boundary contrast of the vessels.

In the last two decades, a large number of methods for vessel segmentation have been proposed, including pattern recognition [1, 2], tubular structure [3, 4] and centerline based approaches [5–7], and active contour model (ACM) [3, 8–10]. Two comprehensive reviews can be referred to in [11, 12]. Among these approaches, ACMs have been widely applied in medical image segmentation, and they can be divided into several classes: edge- [13–18], region- [19–24], and higher level knowledge-based models [5, 8, 9, 25–28]. Edge-based models only utilize image gradient to construct a force to direct contours towards boundaries of desired objects, which makes these models sensitive to noise and prone to fail in weak boundary detection.

Region-based models identify each region of interest by using region statistical information as constraints to guide the motion of the active contour. The most popular region-based ACM is the C-V model [19], and it identifies object and background regions by using global region statistical information. This model has a strict assumption that image intensities are homogeneous in each region; however, such an assumption does not always hold in practice. To address this problem, local region intensity information is incorporated into the energy function [29–36]. For example, some popular ACMs such as the localized active contour (LAC) model [29] and the local binary fitting (LBF) model [35, 36] exactly use local region information as constraints. However, using only local region information makes these models sensitive to noise and the setting of the initial contour. In [37], a hybrid model that combines the local and global statistical intensity information was proposed. This model can reduce the sensitivity to noise and is less sensitive to the initial contour in a way.

Knowledge-based models incorporate some essential features to segment specific objects. Yan and Kassim [8] proposed a capillary active contour that simulates the capillary action that liquid climbs along the boundaries of thin tubes. Lorigo et al. [9] proposed a curve model to regularize the evolution of a surface in a 3D space using the curvature of a line curve instead of the mean curvature of the surface that tends to annihilate vascular structures. The evolution equation is obtained by projecting an auxiliary vector derived from the gradient and the Hessian matrix of the image to the normal plane of the curve. Sun et al. [38] proposed an active contour model based on local morphology fitting for segmenting vessels from 2D angiogram images. These models cannot deal with thin vessels with weak edges. Shang et al. [25] proposed the vascular active contour (VAC) model, where a region competition-based ACM exploiting the double Gaussian mixture model was employed to segment thick vessels, and a vascular vector field (VVF) was implemented to deal with thin and weak vessels. However, this mixture model assumption of the VAC model is unreasonable for some images including time-of-flight magnetic resonance angiography (TOF-MRA) cerebrovascular images, especially for vessel images with intensity inhomogeneity.

This paper proposes a vessel active contour model based on the local intensity weighting and the vascular vector field. The contributions are as follows. (a) An energy function we define is evaluated by local region information, and the function value at each point on the curve is based on the weighted means of interior and exterior in a local neighborhood of the point, which is good for dealing with the intensity inhomogeneity. (b) A vascular vector field (VVF) derived from a vesselness measure is employed to push or pull the evolving contour to locate boundaries of thin and weak vessels. (c) An automatic initialization method based on the vessel shape enhancement is developed, and it can provide a good initial evolution contour that makes the model converge rapidly, which avoids repeated trails in conventional local region active contour models. (d) A speed-up strategy for implementation is developed by labeling the steady points in evolving, which avoids the repeated computation of these points in the subsequent iterations.

The rest of the paper is organized as follows.  Section 2 discusses related works.  Section 3 describes the proposed methodology and its numerical implementation. In Section 4, the proposed method is validated using experiments on synthetic and real images, and comparisons with the LAC, LBF, and VAC models are presented.  Section 5 concludes the paper.

## 2. Related Works

**Figure 1:**
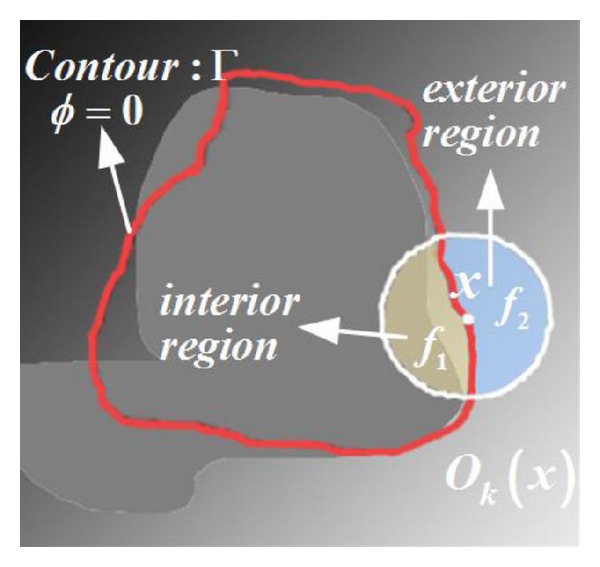
Graphical representation of a local region *O*_*k*_(*x*). The white closed circle *O*_*k*_ denotes the circular neighborhood of *x*. The red closed curve denotes the evolving contour (*ϕ* = 0). The mean intensities of the subregions {*ϕ* < 0}∩*O*_*k*_ (yellow region) and {*ϕ* > 0}∩*O*_*k*_ (blue region) are denoted by *f*_in_ and *f*_out_, respectively.

The key idea of the LAC model is to use local rather than global image statistics to evolve a contour in the variational framework. This model can accommodate variations in intensities that occur over the length of vessels and respond naturally to vessel branches. Lankton [29] directly used this model to segment the coronary artery without any special schemes. The model forms a geodesic energy from local regions around the evolution curve. Let Ω ⊂ *R* ^2^ be the image domain; *I* : Ω → *R* is a given gray level image, and Γ denotes a closed contour represented as the zero level set of a signed distance function *ϕ*; that is, Γ = {*x*∣*ϕ*(*x*) = 0}. For each point *x* on the contour Γ, a local region *O*_*k*_(*x*) centered at *x* is defined and is divided into interior and exterior regions by the active contour, as illustrated in Figure 1.

By virtue of a characteristic function *B*(*x*, *y*), whose value is 1 when the point *y* lies in the local region *O*_*k*_(*x*) or 0 for others, an energy function is defined in terms of a generic internal energy functional *F* as follows:

(1) E(ϕ)=∫Ωxδ(ϕ)∫ΩyB(x,y)·F(I,ϕ,x,y)dy dx,

where *ϕ* denotes the level set function; *δ*(*ϕ*) is the Dirac function to specify the evolving curve; and *F* denotes local adherence to a given model at each point along the contour, and it relies on the simple fitting mean intensities, *f*_in_(*x*) and *f*_out_(*x*), of the local region. In the fitting intensities *f*_in_(*x*) and *f*_out_(*x*), all points in the local region *O*_*k*_(*x*) have an equal contribution. But in fact, the points near the center point *x* should play a more important role than those far away from it. This may affect the segmentation accuracy of some complex objects such as cerebral vessels and carotid vessels.

Li et al. [35, 36] proposed the LBF model by embedding a local binary energy with a kernel function into the total energy. Different from the LAC model, the fitting intensities *f*_1_(*x*) and *f*_2_(*x*) in the LBF model not only take into account the intensities of the points near the central point *x*, but also consider the distances from these points to the central point. This model can segment vessel images more accurately when the initial contour is set appropriately. However, it is worth noting that there are four convolutions to be computed for all pixels of the whole image domain during each iteration in the implementation. Therefore, the computational cost is rather high. Similar to the LAC model, the LBF model requires an appropriate setting of the initial contour and suffers from tending to trap into the local minimum of its energy.

By adding the VVF into the region competition based active contour model, Shang et al. [25] proposed the VAC model for vessel extraction in medical images. In this model, thick vessels are distinguished from background by the region competition based active contour model, and the VVF is employed to cope with weak vessels through the vesselness measure. In the region competition based active contour model, the statistical distributions of the vessel and background are estimated via a Gaussian mixture model. But not all vessel images in real applications can be modeled by the above Gaussian mixture model regarding the intensity distribution. Moreover, the likelihood ratio of the vessel and background may fluctuate substantially near vessel boundaries due to the strong intensity variation. As a result, the zero level set can oscillate intensively on vessel edges, which may lead to an unacceptable segmentation.

The LAC, LBF, and VAC models have their advantages and disadvantages for vessel segmentation. We fully use the advantages of the LAC, LBF, and VAC models in our model. Similar to the LAC model, the energy function we define is evaluated along the evolving curve instead of all image points. Unlike the LAC model, we take into account the contribution difference of different points in the local region when computing the fitting mean intensities *f*_in_(*x*) and *f*_out_(*x*), which is similar to the LBF model. Like the VAC model, we employ a VVF to guide the contour to evolve along the vessel central skeleton into thin and weak vessels. Moreover, an automatic initialization method and a speed-up strategy for implementation are developed, which are superior to the three models.

## 3. Proposed Methodology

In this section, we describe the vessel active contour model based on local intensity weighting and the vascular vector field for vessel segmentation. Localized region energy is defined firstly, and then the VVF based on the vesselness measure is given; next, an automatic contour initialization is presented; at last, a speed-up strategy in implementation is designed.

### 3.1. Localized Region Energy

Inspired by the ideas in [29, 35, 36], we exploit a local region of each point on the active contour instead of the whole image domain to define the energy function. Statistical analysis of the local region can be realized by introducing a kernel function:

(2) Kσ(x,y)=12πσ2e−||x−y||2/2σ2,

where *σ* > 0 is a scale parameter, *x* is the center point, and *y* is the point in the neighbor domain centered at point *x*. In the kernel function *K*_*σ*_(*x*, *y*), the distance between *y* and *x* is fully considered, and *K*_*σ*_(*x*, *y*) decreases and approaches zero with the increase of the distance. Here we use the definition of the localized region energy [35, 36]:

(3) ExLRF(C,f1,f2) =λ1∫in(C)Kσ(x−y)|I(y)−f1(x)|2dy  +λ2∫out(C)Kσ(x−y)|I(y)−f2(x)|2dy,

where *λ*_1_ and *λ*_2_ are the weights of the two integrals over the regions in(*C*) and out(*C*), that is,* interior region* and* exterior region* in Figure 1, respectively. They are usually set as *λ*_1_ = *λ*_2_ to maintain a fair competition between the regions inside and outside of the zero level contour during the evolution. When *λ*_1_ and *λ*_2_ are different, the amounts of penalty imposed on the integrals over in(*C*) and out(*C*) will be different. For example, if *λ*_1_ is larger than *λ*_2_, larger penalty will be imposed on the area of in(*C*). As a result, the emergence of new contour outside the initial contour, which will increase the area of in(*C*), is prevented by a certain degree. In (3), *f*_1_(*x*) and *f*_2_(*x*) are obtained as follows:

(4) f1(x)=∫ΩyKσ(x,y)·Hε(ϕ(y))·I(y)dy∫ΩyKσ(x,y)·Hε(ϕ(y))dy,f2(x)=∫ΩyKσ(x,y)·(1−Hε(ϕ(y)))·I(y)dy∫ΩyKσ(x,y)·(1−Hε(ϕ(y)))dy,

where *H*_*ε*_(*x*) is the Heaviside function used to specify the interior of Γ:

(5) Hε(x)={1,x>ε,0,x<ε,[1+2arctan(x/ε)/π]2,otherwise.

Then the contribution of the intensity *I*(*y*) to the localized region energy *E*_*x*_^LRF^ at the point *x* decreases and approaches zero as the point *y* becomes farther and farther from the center point *x* because of the localization property of the kernel function. As a result, the energy *E*_*x*_^LRF^ is dominated by the intensities of the points in a neighborhood of the center point. In this study, we uniformly choose *σ* = round((dim⁡*y* + dim⁡*x*)/(2 × 8)), where dim⁡*y* and dim⁡*x* denote the row number and the column number of the image, respectively.

Different from the LBF model, we only consider the points on the evolving contour instead of all points in image domain. So the total localized region energy is given by

(6) EΓ=∮Γ(s)Ex(s)LRF(Γ,f1(x(s)),f2(x(s)))ds.

Here a length term |Γ| is added by penalizing the arc length of the curve to keep the curve smooth. The total energy function is defined as follows:

(7) E=EΓ+v|Γ|=∮Γ(s)Ex(s)LRF(Γ,f1(x(s)),f2(x(s)))ds+v|Γ|.

Then, the corresponding level set formulation can be obtained. To preserve the regularity of the level set function *ϕ*, a level set regularization term *P*(*ϕ*) = ∫_Ω_*x*__(|∇*ϕ*(*x*)|−1)^2^/2*dx* is introduced, which is necessary for accurate computation and the stable evolution. Thus, the level set formulation with the regularization term and the penalty term is written as follows:

(8) E=∫Ωxδε(ϕ(x))∫ΩyKσ(x,y)·(Hε(ϕ(y))(I(y)−f1(x))2+(1−Hε(ϕ(y)))×(I(y)−f2(x))2)dy dx +v∫Ωxδε(ϕ(x))|∇ϕ(x)|dx+μ∫Ωx12(|∇ϕ(x)|−1)2dx,

where *v* and *μ* are weighting parameters and *δ*(*ϕ*) can be obtained using the derivative of *H*_*ε*_ in (5) as follows:

(9) δε(x)=Hε′(x)=1πεε2+x2.

Keeping *f*_1_(*x*) and *f*_2_(*x*) constant, the energy function in (8) is minimized with respect to *ϕ* to obtain the gradient descent flow as follows:

(10) fgLRF=∂ϕ∂t(x)=δε(ϕ(x))∫ΩyKσ(x,y)·δε(ϕ(y))×((I(y)−f1(x))2−(I(y)−f2(x))2)dy dx +vδε(ϕ(x))div⁡(∇ϕ(x)|∇ϕ(x)|) +μ(∇2ϕ(x)−div⁡(∇ϕ(x)|∇ϕ(x)|)).

### 3.2. VVF Based on Vesselness Measure

To make the segmentation more accurate for thin and weak vessels, we embed the vesselness measure information into the ACM. Similar to the VAC model, a VVF is defined to restrain the active contour evolution behavior in the vessel segmentation and to form a force to push or pull the evolving contour to locate vessel boundaries.

For 2D images, with regard to the bright vessels with a dark background, we assume that the eigenvalues of the Hessian matrix **H** are *λ*_1_, *λ*_2_  (|*λ*_1_| ≤ |*λ*_2_|), and the corresponding eigenvectors are ${\vec{v}}_{1}$ and ${\vec{v}}_{2}$, respectively. Frangi et al. [39] pointed out that a pixel belongs to a vessel region with |*λ*_1_| ≪ |*λ*_2_|, ${\vec{v}}_{1}$ indicating the direction along the vessel with minimum intensity variation, and ${\vec{v}}_{2}$ being orthogonal to the vessel direction. A geometric ratio *R*_*B*_ = |*λ*_1_|/|*λ*_2_|, based on the second-order ellipsoid, is defined to account for the deviation from a blob-like structure. *R*_*B*_ is at its maximum for a blob-like structure, and is zero whenever *λ*_1_ ≈ 0 or *λ*_1_ and *λ*_2_ tend to vanish. In addition, *R*_*B*_ is grey-level invariant, and it only captures image geometric information. On the other hand, generally for the vessel images, vessel structures are brighter than the background and occupy a relatively small part of the whole image. Thus the magnitude of the derivatives for background pixels is small. So the measure of second-order structuredness based on image intensities can be defined by $S=\sqrt{\lambda_{1}^{2}+\lambda_{2}^{2}}$. The measure will be low in the background where the eigenvalues are small due to the lack of contrast, while it will become larger in the vessel regions with high contrast because of at least one of both eigenvalues being large. By combining *R*_*B*_ and *S*, the vesselness measure *R*(*x*) can be defined as follows:

(11) R(x)={0,if  λ2>0,e−RB2/2α2·(1−e−S2/(2β2)),otherwise,

where *α* and *β* are weighting factors balancing the influence of *R*_*B*_ and *S*.

The VVF direction $\vec{V}(x)$ is defined in terms of the above vesselness measure *R*(*x*):

(12) V→(x)={v→1,if  R(x)>τ,0,otherwise,

where *τ* is a threshold, and it is used to obtain the direction of the vascular vector field. If *τ* is too large, some important vascular vectors may be ignored. If *τ* is too small, numerous redundant vector information, even the nonvessel vector information, is also obtained. In our experiments, *τ* is set as 0.05.

Due to the diameter variation of the vessels, the vesselness measure *R*(*x*) is only high at a certain scale related to the vessel diameter. So, in order to segment vessels with the different sizes, the VVF is calculated under a multiscale framework; that is, the Hessian matrix is calculated by the second-order Gaussian derivatives at multiple scales *σ*, and the response function is normalized by *σ* ^2^ to extract vessels with different sizes. Thus, the maximum response is selected, and the corresponding vector is considered as the final vector, as follows:

(13) V→(x)={V→σ(x) ∣ Rσ(x)=R(x)},

where

(14) R(x)=max⁡σmin⁡≤σ≤σmax⁡{Rσ(x)}.

Since the direction of the VVF coincides with the direction of the normal of the active contour, $\vec{V}(x)$ can be further modified as follows:

(15) V→(x)={V→(x),if  V→(x)·∇ϕ(x)≥0,−V→(x),if  V→(x)·∇ϕ(x)<0.

The magnitude of the VVF, a speed function *f*_*ε*_(*R*(*x*)) related to *R*(*x*), is defined to evolve efficiently:

(16) fε(R(x))=12[1+2πarctan(R(x)−εε)],

where *ε* is the threshold of the vesselness measure. The function *f*_*ε*_(*R*(*x*)) reaches its highest value quickly when *R*(*x*) ≥ *ε* and reaches 0 quickly when *R*(*x*) < *ε*. It means that the active contour moves with high speed inside thin vessels, slows down on the vessel boundaries, and becomes zero outside the vessel region. Furthermore, the magnitude of the VVF rapidly changes near zero of the vesselness measure, and the smaller the *ε* value is, the faster the *f*_*ε*_(*R*(*x*)) value becomes. However, when *ε* ≤ 0.05, *f*_*ε*_(*R*(*x*)) has a subtle change for different *ε*. Therefore, we set *ε* = 0.05.

As a consequence, the vascular vector field can be added in (10) as a constraint term. Thus the total evolution equation of the proposed model is given by

(17) ∂ϕ∂t(x)=fgLRF+λfε(R(x))|V→(x)·∇ϕ(x)|,

where *λ* is a constant. If *λ* is positive, the VVF term, acting as a shrinkable force, will penalize the arc length of the curve along vessel boundaries and force the evolving contour to shrink to thick vessel boundaries, while the weak vessels may be neglected. On the contrary, if *λ* is negative, the VVF term will restrain the influence of the first term *f*_*g*_^LRF^ on contour evolution and tends to push the contour to extend along the weak vessels.

### 3.3. Automatic Contour Initialization

Local region active contour models are often sensitive to the initial contour and need blind and inefficient repeated trails to find the best initial contour. To this end, the vessel shape information is utilized to set the initial contour automatically. We enhance the vessel structures by the vessel enhancing diffusion to extract the rough vessel boundaries. These rough boundaries are used to initialize the contours, and the level set function is initialized as follows:

(18) ϕ(x,t=0)={−ρ,x∈Ω0−∂Ω0,0,x∈∂Ω0,ρ,x∈Ω−Ω0,

where *ρ* is a positive constant, Ω_0_ is the vessel regions in the image domain, and ∂Ω_0_ is the vessel boundaries. The *ρ* chosen should be larger than 2*ε*, where *ε* is the width in the definition of the regularized Dirac function *δ*_*ε*_ in (9). In this study, *ρ* = 2 is set.

### 3.4. Implementation

In order to improve evolution efficiency, redundant computations should be avoided. Firstly, the narrowband technique is adopted. The analysis of local statistics generates a family of local regions at each point on the curve, which restricts the activity of the active contour within the neighborhood of the object boundaries. This occurrence naturally leads to a narrowband in the numeric implementation. Thus, only some points near the contour, instead of all image points, are considered in each iteration. Secondly, a speed-up strategy is designed to further reduce computations, which is based on labeling the steadily evolved points. All the points on the contour are monitored, and the points that do not move in several successive iterations are regarded as the final evolution result. These points will not be computed in the subsequent iterations, and thus the computation load is reduced, especially for the long contours.

**Figure 2:**
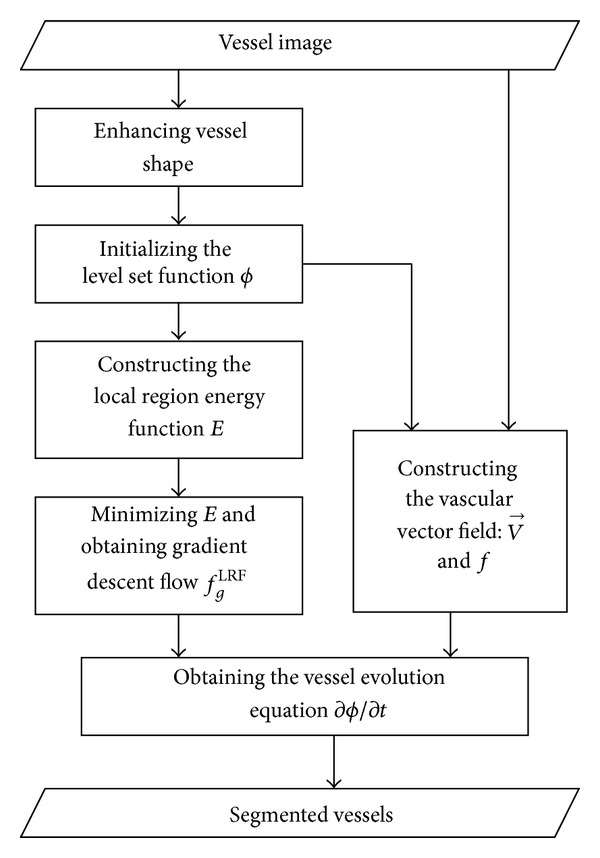
Flow chart for the vessel segmentation of the method.

The flow chart of the proposed method is given in Figure 2. The implementation is straightforward and consists of the following six steps:

1. enhance the vessels and extract the rough boundaries of the vessels;
2. initialize the level set function *ϕ* using (18);
3. update *f*_1_ and *f*_2_ using (4);
4. update $\vec{V}(x)$ and *f*(*R*(*x*)) using (15) and (16):
5. update the level set function *ϕ* using (17);
6. repeat steps (3) to (5) until the convergence criteria are met.

## 4. Experiments and Analysis

Synthetic and real images from different modalities were used to evaluate the proposed model, which was implemented in MATLAB 7.0 on a computer with Intel (R) Pentium (R) Dual 2.0 GHz CPU, 2.0 G RAM, and Windows XP operating system. The same parameters of Δ = 0.1, *v* = 0.2, *μ* = 1.0, and *λ* = −0.1 were used for all the images in this paper, unless noted otherwise.

### 4.1. Synthetic Images

**Figure 3:**
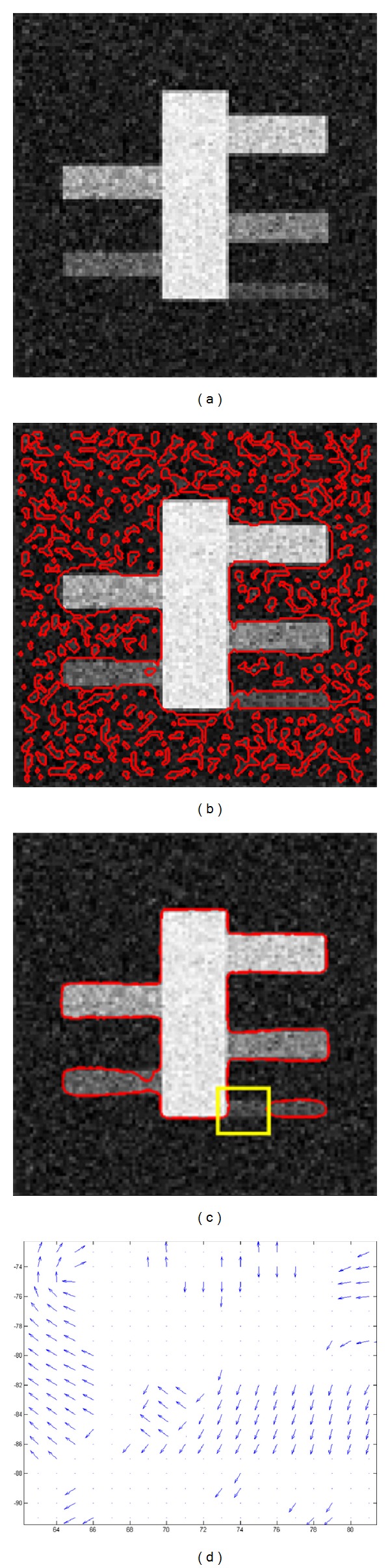
Result of our method for the noise synthetic image. (a) Synthetic image with Gaussian noise; (b) initial contour from our method; (c) final contour after 70 iterations; and (d) VVF corresponding to the local region (marked with the yellow box) in (c).

In order to test the antinoise capability of the proposed model, we used a synthetic noise vessel (SynthNoiseVessel_1) image with 110 × 110 pixels, about 28% Gaussian noise added, as shown in Figure 3(a). All branches of the synthetic vessel have different intensities.  Figure 3(b) shows the initial contour obtained by our method. The final evolving result with 70 iterations is shown in Figure 3(c). We can see that, despite our model does not completely capture the weak boundaries of the bottom branch with strong noise marked with a yellow box in Figure 3(c), it is capable of locating the most vessel boundaries accurately. The result shows that the proposed model is capable of resisting noise to some extent. In addition, the VVF of the local region in Figure 3(c), marked with yellow boxes, was drawn, as shown in Figure 3(d). We can see that the VVF of the image with only the strong Gaussian noise is rather chaotic. In fact, the VVF is well organized for the real vessel images because the blood flow gradually becomes weak from thick vessels to small branches.

### 4.2. Real Images

Experiments were performed using real vessel images with different modalities, including digital subtraction angiography (DSA) images, infrared image, computed tomography (CT) images, and MRA and ultrasonic (US) images of different parts of human body.

**Figure 4:**
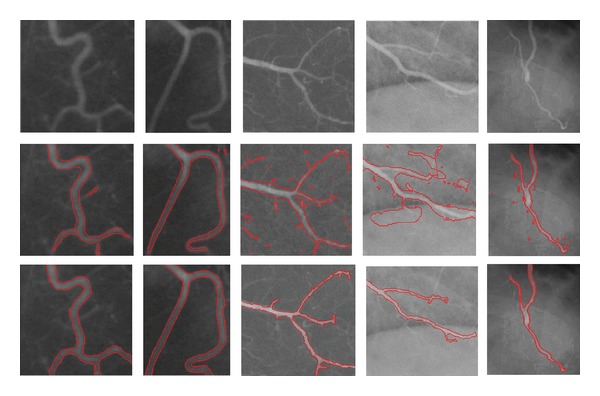
Results of the proposed model for DSA and infrared images. Rows 1, 2, and 3 show the original images, initial contour, and final evolution results, respectively. The image results of the different methods are shown in each column.

Four DSA images and one infrared eye vessel image are shown in the first row of Figure 4 with sizes of 110 × 111, 131 × 103, 211 × 211, 206 × 208, and 211 × 168 pixels, namely, “DSA_1,” “DSA_2,” “DSA_3,” “DSA_4,” and “InfraredEyeVessel,” respectively. The intensities of all these images are inhomogeneous, especially for the right three images. In each column, the original image, the initial contour, and the final evolution result are shown from top to bottom. The results shown in Figure 4 demonstrate that our method is capable of locating the real boundaries accurately for the DSA and infrared vessel images.

**Figure 5:**
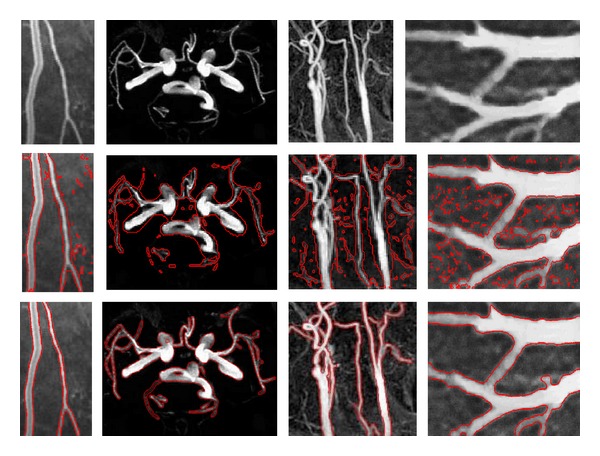
Results of the proposed model for CT, MRA, and US images. Rows 1, 2, and 3 show the original images, initial contour, and final evolution results, respectively. The image results of the different methods are shown in each column.

Figure 5 shows the segmentation results for the CT AbdominalVessel, MRA_CerebralVessel, MRA_CarotidVessel, and US_Plumonary images with sizes of 193 × 102, 202 × 277, 165 × 156, and 138 × 203 pixels, respectively. These original images are shown in the first row from left to right. It can be seen that the shape and topology of the cerebral vessels and carotid images in middle two columns are very complex. The second row shows the initial contours derived from the proposed method based on shape enhancement, and the third row shows the final evolution results. Each column corresponds to the original image, the initial contour, and the final evolution result of the image. The results demonstrate that our method is capable of identifying the real vessel boundaries accurately for the CT, MRA, and US images, although they have complex shape and topology.

**Figure 6:**
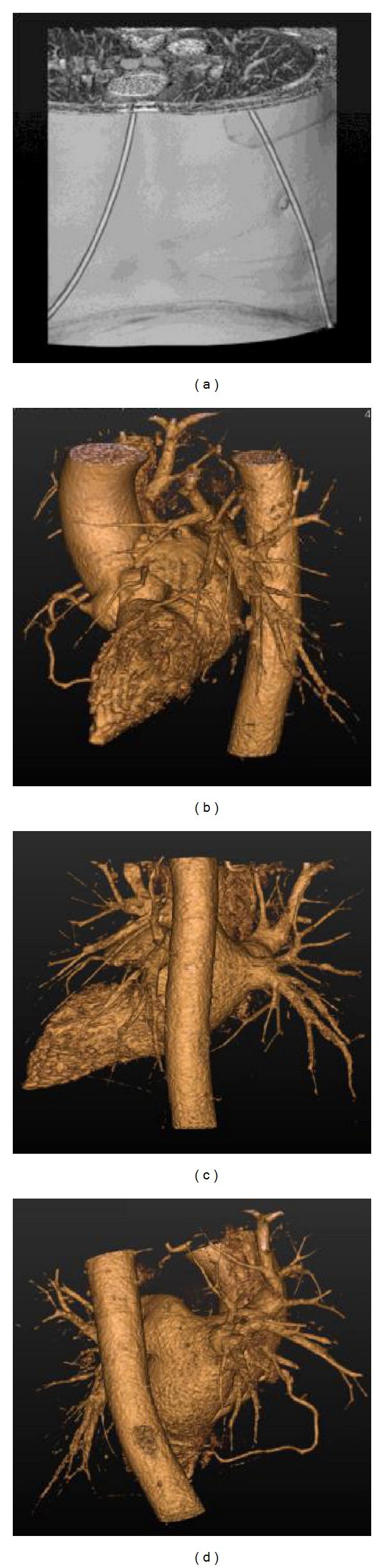
3D original dataset and the extracted vessel trees with different points of view. (a) 3D cardiac CT angiography dataset; (b), (c), and (d) cardiovascular trees from three different points of view.

In addition, we also extended the proposed model to segment the 3D coronary vessel tree. The used data is a 3D cardiac CT angiography dataset with the size of 512 × 512 × 311 voxels.  Figure 6 shows the original dataset and the reconstructed vessel trees with different points of view. It can be seen that different sizes of branches in the vessel trees are extracted.

### 4.3. Comparisons with LAC, LBF, and VAC Models

In this section, our model was compared with three classical models: the LAC, LBF, and VAC models. For comparison, the same initial contour was set for all models.

**Figure 7:**
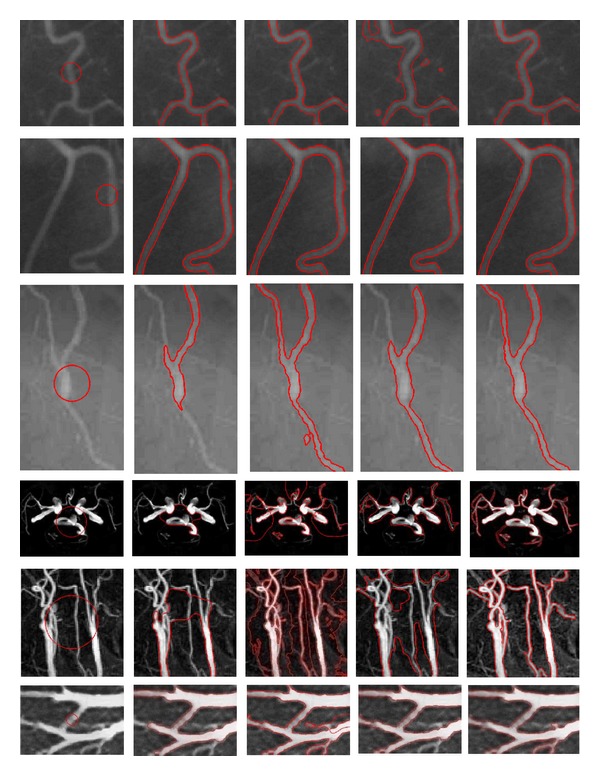
Comparison of our method with the LAC, LBF, and VAC models. Column 1 shows the initial contour for the different images. Columns 2, 3, 4, and 5 show the evolutional results of the LAC model, LBF model, VAC model, and our method, respectively. The image results of the different methods are shown in each row.

Figure 7 shows the results of six real vessel images from Figures 4 and 5. The first column shows the initial contours with different circles. The second column shows the corresponding evolution results of the LAC model, wherein the LAC model cannot completely and accurately locate the vessel boundaries. The reason is that all points in the neighborhood region have the same contribution to the fitting mean intensities, and the distance between the points in the neighborhood region and the center point is not considered. Furthermore, there is lack of a force pushing the contour to evolve along the centerline of weak vessels, which makes the energy function tend to trap into a local minimum. The third column shows the corresponding evolution results of the LBF model. It can be seen that the LBF model is capable of locating the vessel boundaries accurately and can segment the vessels perfectly in the second and third rows. However, the model fails to segment vessels in the other rows. The reason is that the LBF model is sensitive to the initial contour, and the model usually requires setting different initial contours for the disconnected objects. Furthermore, the initial contour needs to be set inside the segmented object or to be intersected with it. The fourth column shows the corresponding results of the VAC model, which are unacceptable. It is because the VAC model strongly depends on the intensity statistical model, and oversegmentation or undersegmentation usually happens when the statistical distribution of a vessel image cannot be modeled appropriately by the Gaussian mixture model. The last column is the results of the proposed method, and it can be seen that our model successfully segments vessels in different images. This illustrates that the proposed method is not sensitive to the contour initialization; that is, it can also locate the vessel boundaries accurately when the initial contour is set in the same manner as that of the conventional models.

**Figure 8:**
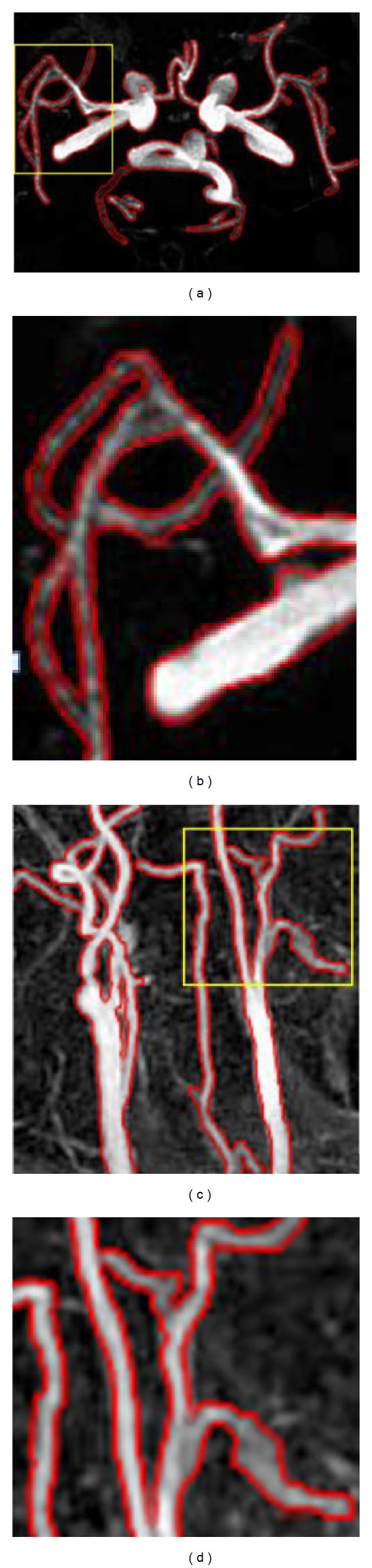
Amplified local regions of the results of our model for the two MRA images. (a), (c) The evolved results for the cerebral vessels and the carotid vessels. (b), (d) The amplified spatial details corresponding to the local region (marked with the yellow box) in (a) and (c), respectively.

Figure 8 shows the amplified local regions of the evolved results of our model for the two MRA images. The local regions marked with yellow boxes are shown in Figures 8(a) and 8(c), and their corresponding amplification results are shown in Figures 8(b) and 8(d), respectively. The amplified local regions present more spatial details. We can see that our model is capable of locating the vessel boundaries accurately in spite of its complex shape and topology.

Modified root mean squared error (MRMSE) [37] was employed to quantitatively evaluate the different methods. MRMSE measures the distance between the exact object boundary and the final contours of the models as follows:

(19) $$\begin{array}{c} \text{MRMSE}=\sqrt{\frac{\sum_{i=0}^{k_{1}-1}\left[{\left(x_{i}-{\overset{-}{x}}_{i}\right)}^{2}+{\left(y_{i}-{\overset{-}{y}}_{i}\right)}^{2}\right]+2\left(k_{2}+k_{3}\right)r^{2}}{k_{1}+k_{2}+k_{3}}}, \end{array}$$

where (*x*_*i*_, *y*_*i*_)  (*i* = 0,…, *k*_1_ − 1) denotes the coordinates of the points on the truth contour; $\left({\overset{-}{x}}_{i},{\overset{-}{y}}_{i}\right)\;\;(i=0,\ldots ,k_{1}-1)$ is the matching point on the evolution contour having the closest distance from point (*x*_*i*_, *y*_*i*_) in the corresponding neighbor window with the size of (2*r* + 1) × (2*r* + 1); *k*_1_ denotes the number of such matching point pairs; *k*_2_ denotes the number of the points on the truth curve without a matching point on the evolution contour; and *k*_3_ denotes the number of points on the evolution contour without a matching point on the truth curve.

The MRMSE values of the evolutional results in Figure 7 are listed in Table 1, and the corresponding window radii were chosen as 12 uniformly. From Table 1, we can see that the MRMSE values of the proposed model are overwhelmingly less than the ones of the other models. It is due to our method's ability to faultlessly draw the advantages of the localized idea of the LAC model, the local energy definition of the LBF model, and the VVF idea of the VAC model. Although the LBF model also has a low MRMSE for several images, the local intensity mean it adopts does not provide enough information for accurate segmentation in the presence of intensity inhomogeneity, for example, the results of two MRA images shown in the 4th and 5th rows of Figure 7.

The CPU time of the different methods was also tested.  Table 2 presents the computational time and the number of iterations for the results in Figure 7. Iteration numbers of our method and the LAC model are almost equal, but time-cost of our method is much less than that of the LAC model. The reason is that the redundant computation in our method is discarded by adopting narrowband technique and labeling the steadily evolved points. As for the LBF model, it usually requires much more time and iterations to achieve stable results because of the computation of *f*_1_ and *f*_2_ for all points in the image domain. Of course, total time of the LBF model can be also reduced by increasing the time step, but the evolved accuracy may be low. As for the VAC model, its time-cost partly depends on whether or not the statistical distribution of intensities can be accurately modeled by a Gaussian mixture model. Unfortunately, the statistical distribution adopted by the VAC model is not suitable for some vessel images. In brief, for almost all images, our model has the lowest both time-cost and iterations, which shows that the implementation of our method is more efficient than the other three models in MATLAB.

### 4.4. Analyzing Contour Initialization

**Figure 9:**
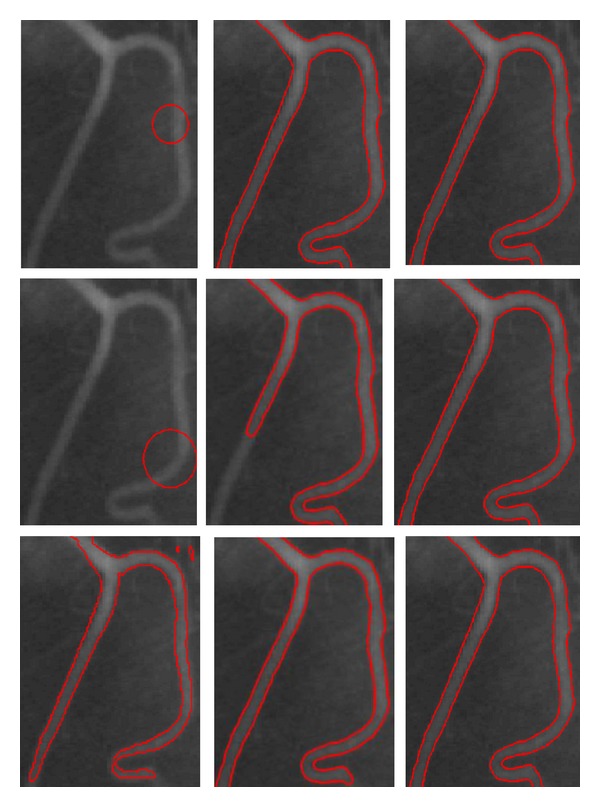
Comparison of the different contour initialization methods for the LAC model and our model. Column 1 shows the initial contours for the DSA_2 image. Columns 2 and 3 show the evolutional results of the LAC model and our method, respectively. The image results of the different initialization contours are shown in each row.

We also compared and analyzed the automatic contour initialization and the conventional manual contour initialization for the conventional LAC model and our model.  Figure 9 shows the evolved results of both methods with different initial contours for the DSA_2 image. The first column shows different initial contours, and the second column and the third column show the corresponding evolved results of the VAC model and our model, respectively. As we can see from the second column that the different results were obtained for the LAC model with three different initial contours, and the active contours stopped to evolve before it did not reach the final location. However, as for our model, the evolved results via the different initial contours are nearly the same, which can be seen from the third column. It demonstrates that our model is not sensitive to the initial contour. This is because the defined VVF can assist and guide the contour to reasonably evolve along the vessel direction, even when the initial contour is not ideal.

In addition, MRMSE, CPU time, and iteration number were computed to evaluate different manners of setting the initial contours in our method. One manner is that used in Figure 7, and the other is the automatic initialization described in Section 3.3.  Table 3 lists the MRMSE, computational time, and the number of iterations needed to process the images in Figure 7. It can be seen that the MRMSE by two manners is comparative, but time-cost and iteration number decrease notably in the automatic manner, which illustrates that our proposed initialization strategy can make the model converge rapidly.

**Figure 10:**
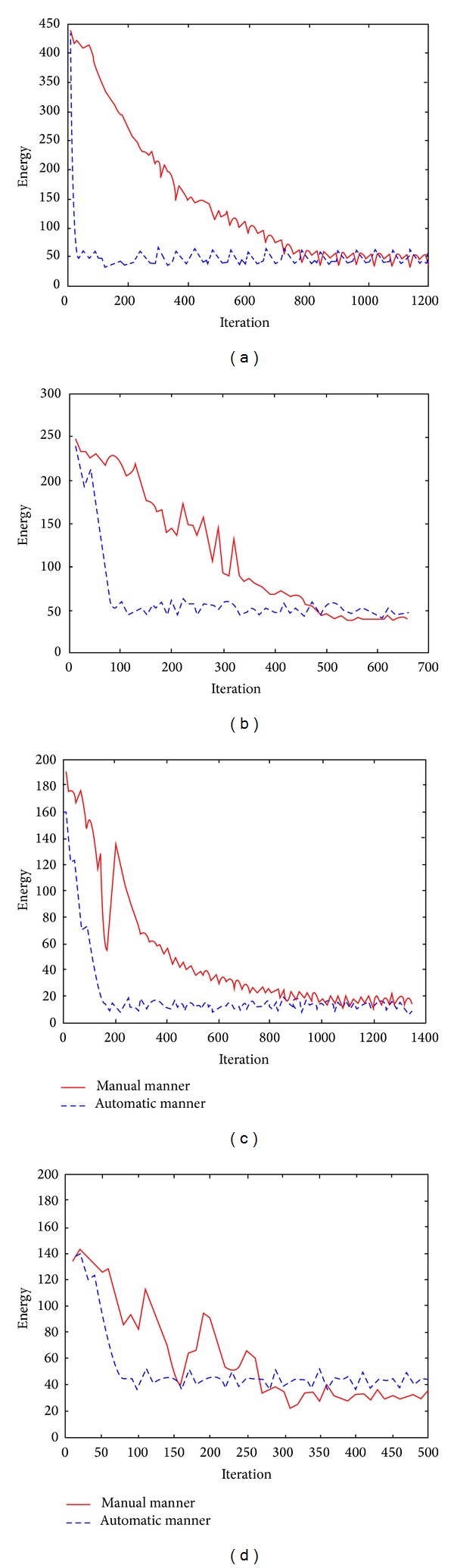
Relationship graphs of energy over iteration of the proposed method with the different initial contours for the four images. (a)–(d) show energy versus iteration for the DSA_1 image, the InfraredEyeVessel image, the MRA_CarotidVessel image, and the US_Plumonary image, respectively.

Figures 10(a) to 10(d) show the energy over iteration of the proposed method with different contour initlization manners, the automatic manner and the manual manner used in Figure 7, for the DSA_1 image, the InfraredEyeVessel image, the MRA_CarotidVessel image, and the US_Plumonary image, respectively. It can be seen that our model using the initial contour set in the automatic manner converges rapidly and smoothly.

## 5. Conclusion

In this study, we presented a vessel active contour model based on local intensity weighting and the vessel vector field. Firstly, a localized energy function, derived from the points on the evolving curve, was defined under the localized active contour model framework. In this definition, the fitting intensity value of the point on the curve was evaluated through a statistical analysis of the interior and exterior weighted means in a local neighborhood of the point, and it was able to deal with the intensity inhomogeneity of the vessels. Secondly, a VVF derived from the vesselness measure was employed to guide the contour to evolve along the vessel central skeleton into the thin and weak vessels. Thirdly, an automatic initialization method for the evolution contour was developed by vessel shape enhancement, which overcame the problem of repeated trials for the initial contour of conventional local region active contour models for vessel segmentation and made the model converge rapidly. Finally, a speed-up strategy in implementation was developed by labeling the steadily evolved points, which avoids the redundant computation of these points in the subsequent iterations.

Extensive experimental results showed the desirable performance of the proposed model. In our future work, we plan to test more 3D volumetric datasets and improve the model to better match to segment 3D vasculatures.

## Figures and Tables

**Table 1:** MRMSE values for the images in Figure 7.

Image name/(row number of Figure 7)	LAC model	LBF model	VAC model	Proposed model
DSA_1/(1th)	12.833	7.0185	19.214	6.4826
DSA_2/(2th)	8.2871	8.7342	10.154	7.9628
InfraredEyeVessel/(3th)	18.965	5.9827	13.644	4.3688
MRA_CerebralVessel/(4th)	121.36	27.466	51.282	14.653
MRA_CarotidVessel/(5th)	90.815	24.927	26.820	11.376
US_Plumonary/(6th)	16.063	10.854	11.501	8.8549

**Table 2:** Iterations and CPU time (in seconds) by the LAC model, LBF model, VAC model, and proposed model.

Image name/(row number of Figure 7)	Size (pixels)	LAC model		LBF model		VAC model		Proposed model	
		Iterations	Time (s)	Iterations	Time (s)	Iterations	Time (s)	Iterations	Time (s)
DSA_1/(1th)	111 × 110	1000	59.157	2800	349.16	1900	70.593	800	29.719
DSA_2/(2th)	103 × 131	1600	225.45	2000	258.37	3500	109.84	1350	83.718
InfraredEyeVessel/(3th)	211 × 168	500	34.328	1400	172.83	2200	83.718	550	30.875
MRA_CerebralVessel/(4th)	202 × 277	1000	71.829	500	69.656	1000	129.98	600	24.931
MRA_CarotidVessel/(5th)	165 × 156	1200	39.735	1500	87.094	1200	94.719	1000	28.856
US_Plumonary/(6th)	138 × 203	400	129.74	500	170.16	900	363.09	350	60.144

**Table 3:** Iterations, CPU time (in seconds), and MRMSE by the proposed model with both different manners of initial contours.

Image name/(row number of Figure 7)	Initial contour used in Figure 7			Initial contour set automatically		
	Iter.	*T*. (s)	MRMSE	Iter.	*T*. (s)	MRMSE
DSA_1/(1th)	800	29.719	6.483	220	20.516	6.122
DSA_2/(2th)	1350	83.718	7.936	420	18.797	7.640
InfraredEyeVessel/(3th)	550	30.875	4.369	90	16.828	4.295
MRA_CerebralVessel/(4th)	600	24.931	14.889	130	14.151	14.653
MRA_CarotidVessel/(5th)	1000	28.856	11.742	200	19.620	11.376
US_Plumonary/(6th)	350	60.144	9.010	110	16.772	8.855
